# Retention in Medical Care Among Insured Children with Diagnosed HIV Infection — United States, 2010–2014

**DOI:** 10.15585/mmwr.mm6639a1

**Published:** 2017-10-06

**Authors:** Mary R. Tanner, Tim Bush, Steven R Nesheim, Paul J. Weidle, Kathy K. Byrd

**Affiliations:** ^1^Division of HIV/AIDS Prevention, National Center for HIV/AIDS, Viral Hepatitis, STD, and TB Prevention, CDC; ^2^Epidemic Intelligence Service, CDC.

In 2014, an estimated 2,477 children aged <13 years were living with diagnosed human immunodeficiency virus (HIV) infection in the United States (*1*). Nationally, little is known about how well children with a diagnosis of HIV infection are retained in medical care. CDC analyzed insurance claims data to evaluate retention in medical care for children in the United States with a diagnosis of HIV infection. Data sources were the 2010–2014 MarketScan Multi-State Medicaid and MarketScan Commercial Claims and Encounters databases. Children aged <13 years with a diagnosis of HIV infection in 2010 were identified using *International Classification of Diseases, Ninth Revision, Clinical Modification* (ICD-9-CM) diagnostic billing codes for HIV or acquired immunodeficiency syndrome (AIDS), resulting in Medicaid and commercial claims cohorts of 163 and 129 children, respectively. Data for each child were evaluated during a 36-month study period, counted from the date of the first claim containing an ICD-9-CM code for HIV or AIDS. Each child’s consistency of medical care was assessed by evaluating the frequency of medical visits during the first 24 months of the study period to see if the frequency of visits met the definition of retention in care. Frequency of medical visits was then assessed during an additional 12-month follow-up period to evaluate differences in medical care consistency between children who were retained or not retained in care during the initial 24-month period. During months 0–24, 60% of the Medicaid cohort and 69% of the commercial claims cohort were retained in care, among whom 93% (Medicaid) and 85% (commercial claims) were in care during months 25–36. To identify areas for additional public health action, further evaluation of the objectives for national medical care for children with diagnosed HIV infection is indicated.

National goals for HIV prevention and care include increasing retention in HIV care (*3*). One indicator for assessing progress toward this goal uses laboratory data reported to the National HIV Surveillance System to track retention in care for adults with diagnosed HIV infection; however, children with diagnosed HIV infection are not included in this assessment (*3*,*4*). The Health Resources and Services Administration evaluates retention in care by assessing the frequency of HIV-related medical visits (*2*). Health insurance claims databases contain information about medical encounters and have been used to assess retention in care for adults with diagnosed HIV infection (*5*,*6*). However, little information is available about retention in care metrics for children with diagnosed HIV infection in the United States.

Data from the 2010–2014 MarketScan Multi-State Medicaid and MarketScan Commercial Claims and Encounters databases were analyzed. MarketScan databases contain de-identified, patient-level health data, including inpatient, outpatient, and pharmaceutical services claims. A unique enrollee identifier is assigned to each client, allowing persons to be tracked across different types of data over multiple years (*5*). The MarketScan Medicaid databases include pooled Medicaid data from 6–12 unidentified geographically dispersed states (*5*). MarketScan Commercial claims databases contain medical data from employees and their spouses and dependents who are covered by employer sponsored private health insurance in the United States (*6*).

Two cohorts were defined from the Medicaid and commercial claims MarketScan databases, using the following eligibility criteria: 1) an ICD-9-CM diagnostic billing code for HIV or AIDS in 2010; 2) aged <13 years in 2010; 3) enrollment in the relevant insurance program for ≥10 months out of each 12-month period during months 0–24; and 4) one or more outpatient visits with a physician, nurse practitioner, or physician’s assistant during the first 6 months of the study period. Children with an ICD-9-CM code for HIV infection or AIDS on only one date were excluded. The study period for each subject extended 36 months from the date of the first claim containing an ICD-9-CM code for HIV infection or AIDS. Using a standard metric, retention in care was defined as at least one medical visit in each 6‐month period during months 0–24, with a minimum of 60 days from the first medical visit in the prior 6‐month period to the last medical visit in the subsequent 6‐month period (*2*). During months 25–36, being in care was defined as having at least one medical visit in each 6‐month period with a minimum of 60 days from the first medical visit in the prior 6‐month period to the last medical visit in the subsequent 6‐month period. This clinic-based definition was used to assess medical visits with clinical providers. Medical visits were counted in the analysis if they were associated with a qualifying outpatient visit Current Procedural Terminology (CPT) code and a provider type code indicating a visit with a physician, nurse practitioner, or physician’s assistant or were associated with a consultation CPT code and a provider type code indicating a visit with an infectious diseases specialist. Visits associated with only a facility type code (e.g., acute care hospital) were not counted.

Demographic characteristics were described, and the unweighted proportion of children in each cohort who were retained in care during months 0–24 was determined. Each cohort was further categorized into retained and not retained subgroups, and within each subgroup, the unweighted proportions of children who met the definition for being in care during months 25–36 were determined. Records for persons not enrolled in the relevant insurance program for ≥10 months during months 25–36 were excluded.

Univariate logistic regression analyses were used to determine odds ratios (ORs) and 95% confidence intervals (CIs) to assess associations between available demographic factors and retention in care during months 0–24. Reference groups for regression were the following: male sex; age ≤1 year (compared with all other ages); basis of Medicaid eligibility was child (compared with blind/disabled individual, foster care child, and eligibility status unknown); and, for the Medicaid database, white race (compared with black, Hispanic, and other). Information on race/ethnicity was not available in the commercial claims database.

Cohorts consisted of 163 children from 4,713,171 unique persons in the Medicaid database, and 129 children from 45,239,752 unique persons in the commercial claims database (Table 1). The Medicaid cohort was predominately black (65%) and included equal proportions of males and females and a higher proportion of children aged 6–10 years (37%) (Table 2). The commercial claims cohort also had nearly equal proportions of males and females but similar proportions of children in all age groups. The most common basis of Medicaid eligibility category was child (not a child of unemployed adult or a foster care child). All children in the commercial claims cohort had a relationship to primary beneficiary category of child/other.

**TABLE 1:** Selection criteria for study cohorts of Medicaid and commercially insured children aged <13 years with diagnosed human immunodeficiency virus infection — MarketScan Multi-State Medicaid and Commercial Claims and Encounters databases, United States, 2010

Criteria	No. children	
	Multi-State Medicaid database	Commercial Claims database
Unique persons in the 2010 MarketScan database	4,713,171	45,239,752
Persons aged <25 years with an ICD-9-CM code for HIV or AIDS in 2010*	1,937	3,036
Children aged <13 years in 2010	403	370
Continuously enrolled months 0–24^†^	278	228
Qualifying outpatient visit in first 6 months of months 0–24^§^	198	158
Excluded^¶,^**	35	29
**Total study population**	**163**	**129**

**Abbreviations:** AIDS = acquired immunodeficiency syndrome; CPT = current procedural terminology; HIV = human immunodeficiency virus; ICD-9-CM = *International Classification of Diseases, Ninth Revision, Clinical Modification*.

* ICD-9-CM code for HIV or AIDS: inpatient or outpatient service claims that listed one or more ICD-9-CM diagnostic codes indicating HIV infection (042, V08, or 079.53). For ICD-9-CM code 795.71 (nonspecific serologic evidence of HIV), children were included only if another qualifying ICD-9-CM code was assigned to them over the course of the study period because code 795.71 is sometimes used to designate HIV-exposed infants before loss of maternal antibodies.

^†^ Continuously enrolled was defined as enrolled in the relevant insurance program for ≥10 months out of each 12-month measurement period.

^§^ Qualifying outpatient visit criteria: an outpatient claim with a physician, nurse practitioner, or physician’s assistant in the first 6 months of the study period. A qualifying visit had to meet one of the following criteria: 1) Outpatient Office Visit CPT code 99201, 99202, 99203, 99204, 99205, 99212, 99213, 99214, 99215, G0463, or T1015, *and* Physician, Nurse practitioner or Physician’s Assistant Provider Type code 200–460, 825, or 845, or 2) Consultation CPT code 99241, 99242, 99243, 99244, or 99275 *and* Infectious Diseases Specialist Provider Type code 285 or 448.

^¶^ Medicaid: excluded because 1) ICD-9-CM code for HIV/AIDS on only one date (n = 31) and 2) Implausible Basis of Eligibility code was “adult, not based on employment,” “aged individual,” or “adult unemployed” (n = 4).

** Commercial Claims: excluded because 1) ICD-9-CM code for HIV/AIDS on only one date (n = 27) and 2) Implausible Relationship to Employee code was “employee” or “spouse” (n = 2).

**TABLE 2:** Characteristics of Medicaid and commercially insured children aged <13 years with diagnosed human immunodeficiency virus infection — MarketScan Multi-State Medicaid and Commercial Claims and Encounters databases, United States, 2010

Characteristic	No. (%)	
	Medicaid (N = 163)	Commercial (N = 129)
**Sex**		
Male	81 (50)	66 (51)
Female	82 (50)	63 (49)
Race/Ethnicity*		
White	19 (12)	NA
Black	106 (65)	NA
Hispanic	7 (4)	NA
Other	31 (19)	NA
**Age group (yrs)**		
≤1	32 (20)	29 (22)
2–5	37 (23)	37 (29)
6–10	60 (37)	34 (26)
11–12	34 (21)	29 (22)
**Medicaid (basis of Medicaid eligibility)**		
Blind/Disabled	38 (23)	NA
Foster care child	22 (14)	NA
Child (not child of unemployed adult or foster care child)	92 (56)	NA
Unknown	11 (7)	NA
**Commercial (relationship to primary beneficiary)**		
Child/Other	NA	129 (100)
Dependent relationship unknown	NA	0

**Abbreviation:** NA = not available.

* No race/ethnicity information is collected within the MarketScan Commercial Claims and Encounter database.

During months 0–24, 60% of the Medicaid cohort and 69% of the commercial claims cohort were retained in care (Figure). In the Medicaid cohort, 148 children remained in the study after month 24, and 117 (79%) were in care during months 25–36. Ninety-three percent of children in the retained in care subgroup and 59% of children in the not-retained subgroup were in care during months 25–36. In the commercial claims cohort, 91 children remained in the study after month 24, and 64 (70%) were in care during months 25–36. Eighty-five percent of children in the retained in care subgroup and 32% of children in the not-retained subgroup were in care during months 25–36, although a high rate of loss from the commercial claims cohort occurred after month 24 (Figure).

**FIGURE:**
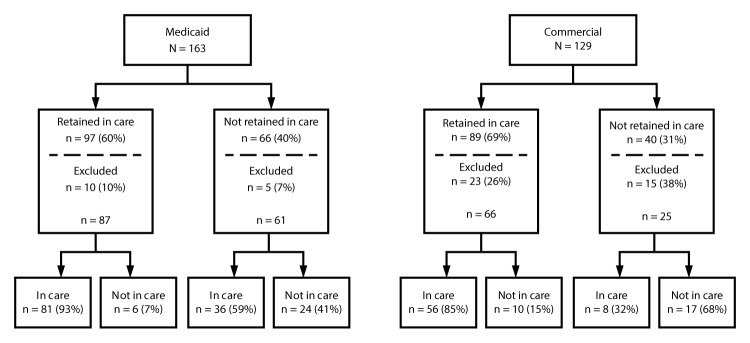
Retention in care* and in-care^†^ status among Medicaid^§^ and commercially^¶^ insured children aged <13 years with diagnosed human immunodeficiency virus infection** — United States, 2010–2014 * Retention in care defined as one or more qualifying outpatient visit in successive 6-month periods over 24 months (months 0–24), with ≥60 days between visits. ^†^ In care is defined as having one or more qualifying outpatient visit during each 6-month period (months 25–36), with ≥60 days between visits. ^§^ MarketScan Medicaid Multi-State databases. ^¶^ MarketScan Commercial Claims and Encounters databases. ** Records of children who were not enrolled in insurance for ≥10 months out of each 12-month period were excluded.

Compared with the reference groups, the basis of Medicaid eligibility categories of blind/disabled and foster child were associated with increased odds of retention in care (OR = 2.45, 95% CI = 1.09–5.53 and OR = 3.40, 95% CI = 1.16–9.99, respectively). In the commercial claims cohort, age ≤1 year was associated with decreased odds of retention in care (OR = 0.38, 95% CI = 0.16–0.88). No other covariates were significantly associated with retention in care in either cohort.

## Discussion

The proportions of children aged <13 years with diagnosed HIV infection who met the standard metric for retention in care for both the Medicaid and commercial claims cohorts were similar to those described in analyses conducted with insurance claims data for adults with diagnosed HIV infection (*5*,*6*). Among the 148 children in the Medicaid cohort after month 24, 117 (79%) were in care during months 25–36, including 59% of children who were not retained in care during months 0–24. This finding illustrates that failure to meet the retention in care definition (i.e., at least one medical visit in successive 6-month periods over 24 months) does not necessarily mean loss to follow-up, although it might suggest gaps in consistency of medical care.

Taken together with national surveillance data that indicate low rates of Stage 3 HIV (AIDS) diagnoses and deaths among children with diagnosed HIV infection (*1*), the need for additional public health attention to pediatric HIV care might not be immediately evident from these results. However, the proportions of children not meeting a retention in care definition based on 6-month interval clinic visits were unexpected in light of the frequency of medical visits recommended in many pediatric HIV care scenarios. U.S. Department of Health and Human Services pediatric HIV treatment guidelines recommend medical assessments every 3–4 months for the first 2 years of antiretroviral therapy (ART), and suggest that there is value in maintaining this frequency for all children with a diagnosis of HIV infection, although some experts might increase the time between assessments for certain stable patients (*7*). Gaps in medical care can result in missed or delayed opportunities for disease prevention (e.g., vaccinations) and might be associated with periods of reduced ART adherence, which could increase the risk for development of antiretroviral resistance (*8*), an issue of particular importance to children with diagnosed HIV infection, given their need for lifelong ART. Overall, the fact that >25% of children with diagnosed HIV infection did not meet the retention in care definition suggests that portions of this medically vulnerable population are not receiving the recommended frequency of medical care.

The finding that a Medicaid eligibility categorization of blind/disabled individual or foster care child was associated with improved odds of being retained in care might be related to increased health care needs and indications for more frequent medical follow-up in both groups (*5*,*9*). The finding that age ≤1 year was associated with decreased odds of retention in care in the commercial claims cohort is surprising, particularly in light of the fact that pediatric HIV treatment guidelines recommend urgent ART and frequent medical follow-up for children < 1 year of age (*7*). This study is not able to identify the reasons for these associations; further investigation into the causes of nonretention in pediatric HIV care is needed. 

The findings in this report are subject to at least five limitations. First, because restricting analyses to HIV primary care visits was not possible, office visits might have been for non–HIV-related issues, which might have caused overestimation of HIV-specific medical care. Second, characteristics of the provider type code used to define outpatient visits could have resulted in an underestimation of retention. Encounters were only counted as visits if they were associated with a provider type code indicating a visit with a physician, nurse practitioner, or physician’s assistant; encounters associated only with the type of facility (e.g., acute care hospital) were not counted. Encounters associated only with facility codes were more common in the Medicaid database. Third, results from the Medicaid and commercial claims cohorts cannot be directly compared because of underlying differences in the databases and the inability to exclude overlap between database populations. Fourth, these findings are based on unweighted proportions and might not be generalizable to the larger population of children with diagnosed HIV infection. Finally, these data do not permit determination of possible causes of nonretention.

The pediatric population with HIV infection has unique needs and challenges. Children’s physiologic maturity and developmental stage affect treatment decisions, and children with HIV infection require close follow-up as they grow and mature (*7*). Children also represent a vulnerable population because they are dependent on their caregivers, and their need for long-term ART makes optimizing medication management important (*10*). The national goal for retention in care for persons aged ≥13 years living with HIV infection, using laboratory tests as a proxy for care visits, is 90% (*3*). Although there is no specific goal for children aged ≤13 years, no reason exists for why children should have a lower retention in care target than adults. Evaluating pediatric retention in care by analyzing results from laboratory testing might provide additional information about retention in care for children with diagnosed HIV infection in the United States. In addition, further investigation into the causes of nonretention in pediatric HIV care is indicated to identify possible areas for public health action.

**Summary**

**What is already known about this topic?**

In 2014, an estimated 2,477 children aged <13 years in the United States were living with diagnosed human immunodeficiency virus (HIV) infection. Guidelines for treating children living with diagnosed HIV infection recommend medical visit frequency of every 3 to 4 months for the first 2 years after care initiation. Nationally, little is known about how well children with diagnosed HIV infection are retained in medical care.

**What is added by this report?**

Applying a clinic-based standard definition of retention in care (≥1 medical visit in successive 6-month periods over 24 months, with ≥60 days between visits), CDC analyzed insurance claims data and estimated that among children with diagnosed HIV infection aged <13 years, 60% of Medicaid-insured children and 69% of commercially insured children were retained in medical care. The retention in care proportions for both cohorts are similar to retention in care findings from analyses conducted with insurance claims data for HIV-diagnosed adults.

**What are the implications for public health practice?**

A substantial proportion of the medically vulnerable population of children with diagnosed HIV infection might not be receiving the recommended frequency of medical care. Further investigation into the causes of nonretention in pediatric HIV care is indicated to identify possible areas for public health action.
